# Surgical Removal of Coronal Fragment of Tooth Embedded in Lower Lip and Esthetic Management of Fractured Crown Segment

**DOI:** 10.5005/jp-journals-10005-1238

**Published:** 2014-04-26

**Authors:** Alok Avinash, Alok Dubey, Rajeev Kumar Singh, Swati Prasad

**Affiliations:** Senior Lecturer, Department of Pedodontics and Preventive Dentistry Rungta College of Dental Sciences and Research, Bhilai Chhattisgarh, India; Reader, Department of Pedodontics and Preventive Dentistry Rungta College of Dental Sciences and Research, Bhilai Chhattisgarh, India; Assistant Professor, Department of Pediatric and Preventive Dentistry, Faculty of Dental Sciences, King George's Medical University, Lucknow Uttar Pradesh, India; Postgraduate Student, Department of Oral Medicine, Rungta College of Dental Sciences and Research, Bhilai, Chhattisgarh, India

**Keywords:** Lower lip, Tooth fragment retrieval, Composite resin reconstruction

## Abstract

Dental fractures of the permanent maxillary anterior teeth are relatively frequent accidents during childhood. The Efficient diagnosis and treatment of dental injury are important elements in clinical dentistry. This article describes a case of trauma in permanent right central maxillary incisors with tooth fragments embedded in the lower lip. Thorough clinical examination followed by soft tissue radiographs confirmed the presence of a fractured incisal fragment, which was surgically retrieved under local anesthesia. Direct composite restoration was placed. After finishing and polishing, an esthetic and natural-looking restoration was achieved; this completely satisfied the functional and esthetic expectation of the patient and dental team.

**How to cite this article: **Avinash A, Dubey A, Singh RK, Prasad S. Surgical Removal of Coronal Fragment of Tooth Embedded in Lower Lip and Esthetic Management of Fractured Crown Segment. Int J Clin Pediatr Dent 2014;7(1):65-68.

## INTRODUCTION

Trauma to teeth is a common situation in a pediatric patient, it may not only damage the dentition but also affect the patient psychologically.^1^ Dental traumatic injuries are a frequent occurrence during childhood, affecting about 13% of the population under 12 years old. Of these fractures, 70% are superior incisor coronal fractures without compromising the root.^2-5^

A number of techniques have been developed to restore the fractured crown. Several factors must be taken into consideration when choosing a treatment for this kind of fracture in a child. Like the teeth are neither totally erupted nor in their final position.^6,7^

The introduction of composite restorative materials in combination with the use of the acid-etch technique to bond composite to enamel made possible the restoration of the fractured incisor with little or no additional tooth pre-paration.^7,8^ The survival rate of repositioned fragments is low after 2 years in case of large fractured fragments.^9^ If the lost fragment is not recovered or it is inadequate for repositioning, it would be advisable to use composite reconstruction. Although composite restorations tend to degrade with time, losing their esthetic properties, they are, however, more resistant long term.^10^

This case report describes a child with traumatic amputation of anterior crown with incisal fragment being embedded in the lower lip. The child was managed by surgical removal and composite reconstruction of crown fracture.

## CASE REPORT

**Fig. 1:**
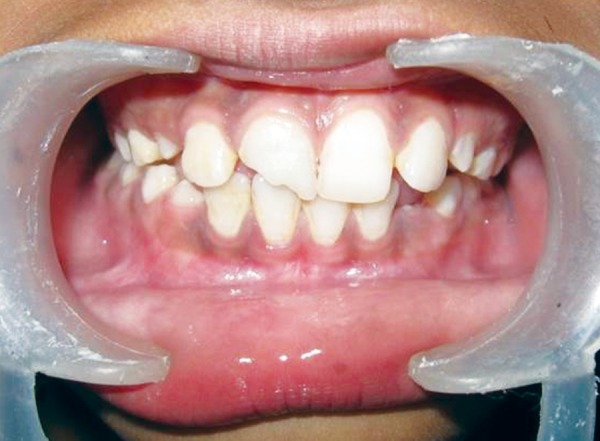
Preoperative view showing fractured permanent right maxillary central incisor and lower lip with no signs of inflammation

**Fig. 2:**
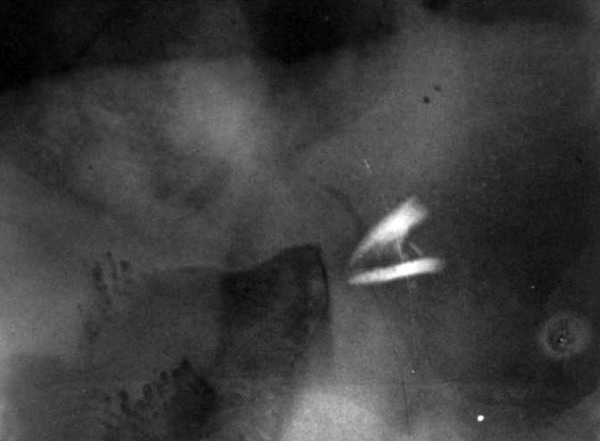
Radiographic image showing tooth fragment in the lower lip

A 10-year-old female patient reported to our department following trauma that caused a fracture in the distal angle of the maxillary right central incisor that affected enamel and dentine with pulp exposure (Fig. 1). Trauma occurred 10 months back due to fall while running on the road. The parents were concerned about the esthetics of the child. On inspection, a swelling on the left side of lip was noticed. A firm nodule measuring approximately 1 cm in diameter in the same region was palpated. Tooth showed no vitality for pulp tests. Radiograph of the lip confirmed the presence of a tooth fragment in the lower lip (Fig. 2).

**Fig. 3:**
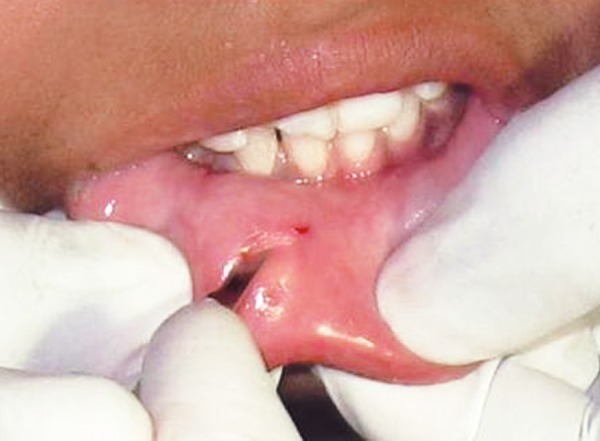
Horizontal incision given on the lower lip

**Fig. 4:**
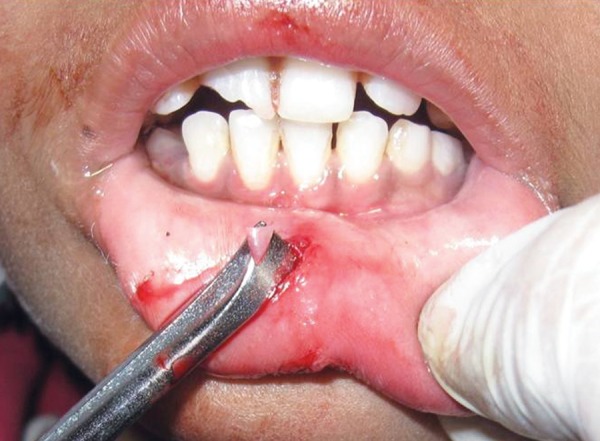
Tissue retracted for the retrieval of the tooth fragment

**Fig. 5:**
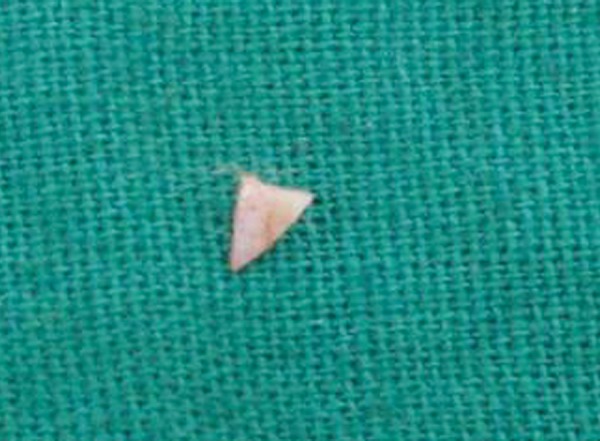
Tooth fragment after its removal from lower lip

The patient was submitted to surgical excision of the fragment under local anesthesia. The lower lip was incised (Fig. 3), tissues were reflected, tooth fragment was located (Fig. 4) and removed carefully (Fig. 5).

**Fig. 6:**
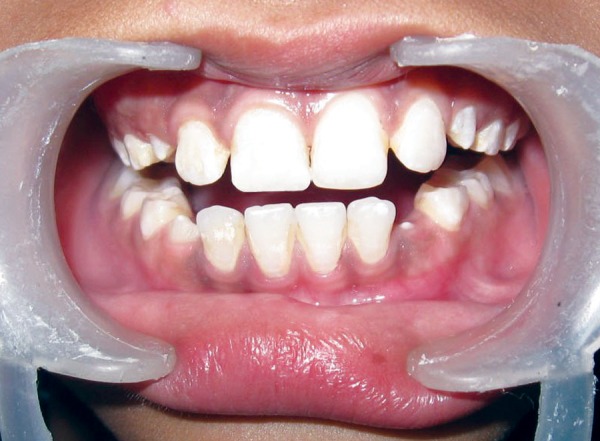
Composite reconstruction of the fractured crown and healed lower lip after 15 days

After placement of a rubber dam, pulp was extirpated; the canal dressed following instrumentation and then obturated. The entrance of the root canal was sealed with glass ionomer cement. Composite reconstruction was planned as long time had elapsed since the tooth had fractured. Initially, color was determined and, for that, in the gingival, middle and incisal areas small quantities of different colors of composite were placed and cured as a color determination method. The colors chosen were A3 for the cervical third and A2 for the middle and incisal thirds (Z 100^TM^, 3M ESPE, St Paul, USA). Using a brush in counter-angle hand piece, the surface was cleaned with pumice stone powder. In addition, an extensive bevel was performed to increase adhesion surface and improve esthetics. Composite reconstruction was done 15 days after surgical removal of the tooth fragment (Fig. 6).

## DISCUSSION

Crown fractures of permanent teeth are common pathology in school ages as nearly half of the children have at least one traumatized tooth before they leave school.^11^ The incidence of anterior teeth crown fractures in the permanent dentition is about 26 to 76%.^12^ Usually, a fractured or missed incisor does not pose any problem in diagnosis. However, when this situation is added to soft tissue laceration, attention should be paid to whereabouts of the fractured fragments of the teeth.^13^Proper radiographic evaluation of the patients who have lost partially or totally their teeth after maxillofacial trauma is extremely important, as they are foreign bodies at risk for ingestion, inclusion in surrounding tissue or aspiration. The worst complication is aspiration of foreign bodies that can lead the patient to a variety of chronic airway problems and even death if not precociously diagnosed.^14,15^

Another important factor is the differential diagnosis, mainly in delayed trauma, because the radiographic image of dental fragments included in the mouth foor can be similar to sialolith of the salivary glands.^15,16^ In the case presented here, the tooth fragment was embedded in the lower lip which was confirmed by the radiograph. The general dentist and even the patient failed to notice the presence of broken tooth fragment. As the healing of the laceration took place, the fragment was covered by fibrous tissue. The treatment of choice in this case was surgical excision. Immediately after excision, the soft tissue radiograph is mandatory to ensure the complete removal of fragments, as failure to remove them totally may lead to breakdown of the suture line, persistent chronic infection, pus discharge and a dis-figuring fibrosis.^17-19^

Despite the fact that the restoration was projected as a temporary treatment whilst awaiting an adequate maxillary growth of the patient, the durability of the treatment confirms that with proper case selection, this kind of treatment may be feasible in the long term.^10^ The attachment of the fractured fragment of the teeth following the sandwich technique, described by Simonsen,^8^ represents a very successful frag­ment repositioning system with excellent esthetic properties. However, carrying out this technique presents crucial diff-culties, including a very demanding dentin casting process and a complex fragment alignment task, particularly at the interproximal level. Recent studies have proved that it presents inferior longevity compared with composite resto-rations.^9^ The functional behavior of a porcelain crown, from an esthetic and mechanical point of view, is superior to a composite,^18,19^ but it is contraindicated in a child at the age of ten, with an immature dental and periodontal system. In addition, composite can be easily repaired, reconditioned or even replaced by porcelain crowns in the future.^20^

## CONCLUSION

This case report emphasis the need for thorough clinical and radiographic examination in all cases of dental trauma especially, soft tissue injury accompanying dental trauma. Every attempt should be made to locate the missing tooth structure through a detailed history of the accident and careful examination.

## References

[1] Robertson A, Robertson S, Noren JG (1997). A restorative evaluation of traumatized permanent teeth.. Int J Paed Dent.

[2] Hamdan MA, Rajab LD, (Faculty of Dentistry, The University of Jordan, Amman. anwarm@ju.edu.jo), (2003). Traumatic injuries to permanent anterior teeth among 12-year-old schoolchildren in Jordan.. Community Dent Health.

[3] Zerman N, Cavalleri G, (Dental Clinic, University of Verona, Italy), (1993). Traumatic injuries to permanent incisors.. Endod Dent Traumatol.

[4] Bastone EB, Freer TJ, McNamara JR, (Dental School, University of Queensland), (2000). Epidemiology of dental trauma: A review of the literature.. Aust Dent J.

[5] Saroglu I, Sonmez H, (Department of Pedodontics, School of Dentistry, Ankara University, Ankara, Turkey), (2002). The prevalence of traumatic injuries treated in the pedodontic clinic of Ankara University Turkey, during 18 months.. Dent Traumatol.

[6] Simonsen RJ (1982). Restoration of a fractured central incisor using original teeth.. J Am Dent Assoc.

[7] Hegde RJ, (Department of Pediatric and Preventive Dentistry, PMNM Dental College and Hospital, Bagalkot, Karnataka). (2003). Tooth fragment reattachment—an esthetic alter-native: Report of a case.. J Indian Soc Pedod Prev Dent.

[8] Burke FJ, (Department of Restorative Dentistry, Turner Dental School, Manchester). (1991). Reattachment of a fractured central incisor tooth fragment.. Br Dent J.

[9] Peumans M, Van Meerbeek B, Lambrechts P, Vanherle G, (BIOMAT, Department of Operative Dentistry and Dental Materials, School for Dentistry, Oral Pathology and Maxillo-Facial Surgery, Catholic University of Leuven, Belgium), (1997). The 5-year clinical performance of direct composite additions to correct tooth form and position. I. Esthetic qualities.. Clin Oral Investig.

[10] Garcia-Ballesta C, Percz-Lajarin L, Cortes-Lillo O, Chiva Garcia F, (The Department of Dentistry, University of Murcia, Spain), (2001). Clinical evaluation of bonding techniques in crown fractures.. J Clin Pediatr Dent.

[11] Andreasen JO, Ravn JJ (1972). Epidemiology of traumatic dental injuries to primary and permanent teeth in a Danish population sample.. Int J Oral Surg.

[12] John R, Prabhu NT, Munshi AK (1998). Reattachment of fracture maxillary incisor crown: A case report.. J Indian Soc Pedo Prev Dent.

[13] Da Silva AC, De Moraes M, Bastos EG, Moreira RWF, Passeri LA, (Private Practice, Santos city, Brazil), (2005). Tooth fragment embedded in the lower lip after dental trauma: Case reports.. Dent Traumatol.

[14] Shetty RM, Sholapurkar AA, Dixit U (2010). Rev Clín Pesq Odontol Curitiba.

[15] Kimberly DR, (Department of Oral and Maxillofacial Surgery, William Beaumont Army Medical Center, El Paso, TX 79920-5001, USA. david.kimberly@amedd.mil.com). (2001). Unrecognized aspiration of a mandibular incisor.. J Oral Maxillofac Surg.

[16] Goldstein EJ, (R.M. Alden Research Laboratory, Santa Monica Hospital Medical Center, California). (1992). Bite wounds and infection.. Clon Infect Dis.

[17] Chu FC, Yim TM, Wei SH, (Faculty of Dentistry, University of Hong Kong, Hong Kong. cschu@hkucc.hku.hk), (2000). Clinical consideration for reattachment of tooth fragments.. Quintessence Int.

[18] Belcheva A (2008). Annual Proceeding (Scientific Papers),. Journal of IMAB.

[19] Palmqvist S, Swartz B, (Department of Prosthetics, Postgraduate Dental Education Center, Orebro, Sweden), (1993). Artificial crowns and fixed partial dentures 18 to 23 years after placement.. Int J Prosthodont.

[20] de la Peña VA, Cabrita OB, (School of Dentistry, Faculty of Medicine and Dentistry, University of Santiago de Compostela, Spain. victorap@mundo-r.com), (2005). Direct composite coronal reconstruction of two fractured incisors: An 8-year follow-up.. Dent Traumatol.

